# The impact of DO and salinity on microbial community in poly(butylene succinate) denitrification reactors for recirculating aquaculture system wastewater treatment

**DOI:** 10.1186/s13568-017-0412-3

**Published:** 2017-06-02

**Authors:** Ya-Le Deng, Yun-Jie Ruan, Song-Ming Zhu, Xi-Shan Guo, Zhi-Ying Han, Zhang-Ying Ye, Gang Liu, Ming-Ming Shi

**Affiliations:** 1 Institute of Agricultural Bio-Environmental Engineering, College of Bio-systems Engineering and Food Science, Zhejiang University, 866 Yuhangtang Road, Hangzhou, 310058 China; 2 Aquaculture and Fisheries Group, Department of Animal Sciences, Wageningen University, 6708 WD Wageningen, The Netherlands; 3 Department of Biological and Environmental Engineering, Cornell University, Riley Robb Hall, Ithaca, NY 14853 USA

**Keywords:** Recirculating aquaculture system, Biological denitrification, Salinity, Dissolved oxygen, Microbial community, Real-time PCR

## Abstract

The interactions between environmental factors and bacterial community shift in solid-phase denitrification are crucial for optimum operation of a reactor and to achieve maximum treatment efficiency. In this study, Illumina high-throughput sequencing was applied to reveal the effects of different operational conditions on bacterial community distribution of three continuous operated poly(butylene succinate) biological denitrification reactors used for recirculating aquaculture system (RAS) wastewater treatment. The results indicated that salinity decreased OTU numbers and diversity while dissolved oxygen (DO) had no obvious influence on OTU numbers. Significant microbial community composition differences were observed among and between three denitrification reactors under varied operation conditions. This result was also demonstrated by cluster analysis (CA) and detrended correspondence analysis (DCA). Hierarchical clustering and redundancy analysis (RDA) was performed to test the relationship between environmental factors and bacterial community compositions and result indicated that salinity, DO and hydraulic retention time (HRT) were the three key factors in microbial community formation. Besides, *Simplicispira* was detected under all operational conditions, which worth drawing more attention for nitrate removal. Moreover, the abundance of *nosZ* gene and 16S rRNA were analyzed by real-time PCR, which suggested that salinity decreased the proportion of denitrifiers among whole bacterial community while DO had little influence on marine reactors. This study provides an overview of microbial community shift dynamics in solid-phase denitrification reactors when operation parameters changed and proved the feasibility to apply interval aeration for denitrification process based on microbial level, which may shed light on improving the performance of RAS treatment units.

**Electronic supplementary material:**

The online version of this article (doi:10.1186/s13568-017-0412-3) contains supplementary material, which is available to authorized users.

## Introduction

Nitrate in the wastewater is toxic and can cause sever eutrophication to the environment with the increasing discharge of high nitrate concentration wastewater (Wang and Chu 2016). Among various nitrate removal methods, biological denitrification seems to be one of the most promising processes due to its flexibility and high cost efficiency, and heterotrophic denitrification was the most common selection as its high nitrate removal rate (Ashok and Hait 2015). During the process, nitrate is reduced to nitrogen gas by heterotrophic bacteria with nitrite, nitric oxide and nitrous oxide as intermediate products, which are usually catalyzed by four enzymes coded by gens *Nar*, *Nir*, *Nor* and *Nos* step by step with organic materials utilized as the electron donor (Zumft 1997). Because of the special water quality characteristic of low C/N ratio in such water body as ground water, drinking water or recirculating aquaculture system (RAS), additional liquid organic substance is needed as external carbon source in traditional denitrification system, which requires sophisticated control of dosage and continuous monitoring (Sauthier et al. 1998). Recently, an interesting alternative of solid-phase heterotrophic denitrification process was proposed, in which insoluble solid substances were used as the carbon source and biofilm carrier to remove nitrate from RAS wastewater (Boley et al. 2000). During last decades, various biodegradable polymers (BDPs) including polyhydroxyalkanoates (PHAs) (Hiraishi and Khan 2003), polycaprolactone (PCL) (Boley and Muller 2005), polylactic acid (PLA) (Fan et al. 2012) and poly(butylene succinate) (PBS) (Zhu et al. 2015) were utilized for various kinds of wastewater treatment and achieved acceptable denitrification performance.

It is well known that the variations in operational parameters and environmental factors affect the ability of bacteria to biologically transform N compounds (Kim et al. 2011). In previous studies, the influential factors in solid-phase denitrification were mainly focused involving temperature, dissolved oxygen (DO) and HRT (hydraulic retention time) (Wang and Chu 2016). Traditionally, denitrification process is mainly performed under anaerobic conditions because oxygen, as a more energetically efficient electron acceptor, can highly inhibit the activities of denitrification enzymes and thus cause nitrite or nitrous oxide accumulation (Jia et al. 2013). However, conflict denitrification performances were found in previous solid-phase denitrification with the present of DO. The negative effects were reported as the DO concentration increase in polyhydroxybutyrate (PHB) denitrification (Gutierrez-Wing et al. 2012), while higher nitrate removal rates were obtained in PBS denitrification reactors under similar circumstances (Luo et al. 2014; Ruan et al. 2016). On the other side, the study related to the factor of salinity is still limiting, which only mentioned in PHB or PBS denitrification performance (Gutierrez-Wing et al. 2012; Zhu et al. 2015). In general, salinity was thought a stressor on anoxic denitrification (Miao et al. 2015), but enhanced nitrate removal stability and denitrification rate were found in marine PBS denitrification process (Zhu et al. 2015). Therefore, in order to have a better understanding of the BDPs denitrification, it is essential to reveal the difference and connection between microbial community structure and various operation parameters, thus clarify deeply the shift on microbial level out of the superficial denitrification performance variation.

Recent studies have shown that high-throughput sequencing technologies including Illumina sequencing have been considered as promising methods for the metagenomic analysis due to their enough sequencing depth and high accuracy to cover the complex bacterial communities. The high-throughput sequencing technologies have been applied to investigate bacterial community structure and explore denitrification functional genes in denitrification bioreactors (Miao et al. 2015; Qiu et al. 2016). Besides, adequately linking the changes in quantitative composition of bacteria involved in the N-cycle to environmental variables may contribute to further improvements in bioreactor design and operation. In solid-phase denitrification, the microbial community of biofilm attached on various kinds of biodegradable media were studied, and *Diaphorobacter*, *Acidovorax*, *Simplicispira* and *Comamonas* were found as most commonly predominant denitrification bacteria in previous research (Luo et al. 2014; Shen et al. 2016; Wu et al. 2013; Zhu et al. 2015). However, the overall microbial community shift and its relationship with variables was still not clear. Knowledge from comparisons of such bacterial community structures in different operated habitats may benefit to identifying habitat-specific adaptations in its gene expression. It can also reveal whether certain functional groups were responsible single or co-occur for the observed denitrification performance, especially with present of DO and salinity.

In the present study, we aimed to investigate the microbial community structure and diversity shift dynamics under various influents which mainly involving factors of DO and salinity in PBS heterotrophic denitrification process. And the principle environmental factors driving the bacterial community distribution were also further discussed. Moreover, the potential of most dominant bacteria found in this study is also discussed in detail on comprehensive literal review. In addition, the absolute quantification of *NosZ* gene and 16S rRNA were also detected. Therefore, this study may extend our knowledge about the molecular ecological mechanisms of solid phase denitrification processes for further implication.

## Materials and methods

### Description of three different PBS denitrification reactors

The operation conditions and sample numbers in different stages of three denitrification reactors were shown in Additional file 1: Table S1. The Reactor I (salinity, 0‰) and Reactor II (salinity, 25‰) were up flow fixed-bed anoxic denitrification reactors packed with PBS as carbon source and biofilm carrier as described in our previous study (Zhu et al. 2015). The Reactor III was an airlift inner-loop sequencing batch reactor (salinity, 25‰) packed with PBS carriers to the volume of 50%, which was operated under intermittent aeration strategy as introduced in our previous work (Ruan et al. 2016). The component of synthetic wastewater and real tilapia RAS wastewater used as influent were reported in previous study (Zhu et al. 2015). Temperature was controlled by the dark artificial climate room. The inorganic-N (TAN, NO_2_^−^-N, NO_3_^−^-N) concentrations were measured according to standard methods (SEPA 2002). The dissolved organic carbon (DOC) concentrations in both influent and effluent were analyzed using TOC analyzer (Multi N/C 2100, Analytik Jena, Germany), and pH/OPR were detected using a pH meter (Mettler Toledo, Shanghai, China). To study the response of microbial diversities and community structures to varied operation stages, regular sampling of biofilm was conducted according to Additional file 1: Table S1.

### DNA extraction and PCR amplification

The attached biofilms of varied reactors as describe in Additional file 1: Table S1 were sampled from 20 mL PBS granules through ultrasonic treatment for 2 min at a frequency of 53 kHz. DNA extraction was performed by using FastDNA^®^ Spin Kit for Soil (MP Biomedicals, CA, USA). The extracted genomic DNA was quantified using a NanoDrop ND-2000 spectrophotometer (Thermo scientific, Waltham, MA USA) and examined in 1.0% agarose gels by electrophoresis. The primer pairs 341F (5′-CCTACGGGNGGCWGCAG-3′) and 805R (5′-GACTACHVGGGTATCTAATCC-3′) were used to amplify the V3–V4 hypervariable regions of bacterial 16S rRNA as previously described (Herlemann et al. 2011). The PCR amplification of 50 μL reaction system was performed as the following protocol: an initial denaturation step of 3 min at 94 °C, followed by 5 cycles (94 °C for 30 s, 45 °C for 20 s, 65 °C for 30 s) and 20 cycles (94 °C for 20 s, 55 °C for 20 s, 72 °C for 30 s), and a final extension at 72 °C for 5 min.

### Illumina high throughput sequencing and bioinformatics analysis

The PCR amplifications were extracted from 2% agarose gels and purified using the Agencourt^®^ AMPure XP Beads (A63881, Beckman, USA). All the qualified amplifications were paired-end sequenced (2 × 250/300 nt multiplex) on an Illumina MiSeq platform at Zhejiang Institute of Microbiology (Hangzhou, Zhejiang, China) according to the standard protocols and software (Data collection software, Illumina).

After Illumina sequencing, downstream sequence analysis was performed using Mothur software (http://www.mothur.org) based on standard procedure (Schloss et al. 2009). The paired-end reads were joined by PRINSEQ-lite 0.19.5, chimera was detected by Uchime, and low quality and short sequences were then removed. Afterwards, the resulting high quality sequences were processed to generate operational taxonomic units (OTUs) by ribosomal database project (RDP) at the 97% sequence similarity threshold. Besides, the alpha microbial biodiversity of the 15 samples was estimated by calculating Chao1 richness estimator, and indexes of ACE (abundance-based coverage estimator) and Simpson. Moreover, the coverage and the rarefaction curves were also calculated.

To compare the composition of bacterial communities within and between each denitrification samples, cluster analysis (CA) and principal component analysis (PCA) of microbial structures were conducted using Paleontological statistics software (PAST, version 3.01) based on the algorithm of Bray–Curtis at the genus level. Detrended correspondence analysis (DCA) was used to estimate microbial community composition heterogeneity. Since the first gradient axes length was 2.672, redundancy analysis (RDA) were used to evaluate the ecological distribution of the bacterial communities and their correlation with environmental factors by CANOCO software (Braak and Smilauer 2002).

### Abundance of 16S and *nosZ* genes in the PBS-reactors by quantitative real-time PCR

To quantitatively analysis the denitrifiers and total bacteria cells, real-time PCR was conducted to amplify the denitrifying gene of *nosZ* and 16S rRNA within the last DNA samples (Additional file 1: Table S1, R1-D*, R2-D*, R3-C*) collected in the middle of all three reactors. The primers, reaction systems and PCR programs are listed in Additional file 1: Table S2. All quantitative amplifications were conducted in triplicate using the SYBR Green Real-Time PCR Kit (Novland, Shanghai, China) and respective primers on Mx3000P qPCR System (Agilent, Germany). In detail, the PCR product of each functional gene was purified by using the QIA-quick^®^ Gel Extraction Kit (Qiagen). Plasmids carrying target fragment were then extracted and purified by using the Axygen mimi-prepare (Axygen), and the concentration was measured by using micro spectrophotometry (NanoDrop ND-2000, Willmington, USA). The RT-PCR mixture (25.0 μL) contained 12.5 μL of SYBR Premix Ex Taq Super Mix (TaKaRa Japan), 0.5 μL of each primer (10 μM), 1.0 μL of template DNA (10 ng/μL) and 10.5 μL of ddH_2_O. DNA cloning was used to construct recombinant plasmids, and five to seven-point calibration curves (Ct values versus log of initial target gene copy) were generated using tenfold serial dilution of plasmids for absolute quantification. In order to calculate the absolute abundance of each target gene, the number of gene copies was compared to the amount of the extracted DNA. The reaction efficiency of standard curves for *nosZ* and 16 sRNA genes were 100 and 97% respectively with R^2^ values of 0.99. As a useful approach to estimate the gene activity of the denitrifiers and bacterial community, the number of *nosZ* gene copies was expressed as bacteria cell numbers. It should be noticed that the average number of 16S rDNA copies per bacterial cell is 4.2, and the *nosZ* gene most often carry a single copy (Palmer et al. 2009). Furthermore, one way ANOVA analysis of the RT-PCR results was performed using IBM SPSS statistics 20.0 for Windows (IBM Corporation, NY, USA) where the differences were considered significant when P < 0.05.

### Accession numbers

The sequences were deposited in GenBank under Accession Number SRR3883477-SRR3883491.

## Results

### Overview of sequencing and microbial diversity shift

The microbial community diversities of three PBS denitrification reactors under varied operational conditions were investigated by Illumina Miseq sequencing. At the taxonomic level 373,246 16S rRNA high-quality gene sequences were acquired from 15 samples of the three reactors; the number of reads ranged from 21,849 to 29,103 per sample with the coverage range from 97.81 to 99.33%. Rarefaction curves showed enough sequencing depth was conducted though several minor species remained unidentified (Additional file 1: Figure S1). Subsequently, a total of 3508 OTUs were generated at the 97% similarity level. The unique OTU numbers of 15 samples and shared OTU percentage among each reactor were shown in Table 1. Reactor I had the richest diversity (OTU, 644-901), followed by Reactor II (OTU, 466-534) while samples from Reactor III had only 332-473 OTUs and showed the lowest diversity. Furthermore, an increased trend of OTU numbers was only detected in Reactor I, while in other two reactors the OTU numbers were relatively stable. The results of ACE, Chao 1, Shannon indices indicated the similar trends with the OTU numbers. For each community, the Shannon indexes (H) were in the range of 2.04–3.62 (lower H values, lower α-diversity), corresponding to the low OTU values ranging from 332 to 901, and Chao1 values from 579 to 2146 (Table 1).

**Table 1:** The unique OTU numbers, percentages of shared OTU and α-diversity of three treatment PBS reactors

	R1				
	R1-A	R1-B	R1-C	R1-D	R1-D*
R1-A	***707***	*25.52*%	*18.67*%	*13.80*%	*12.38*%
R1-B		***739***	*27.15*%	*19.80*%	*18.60*%
R1-C			***755***	*23.77*%	*25.13*%
R1-D				***901***	*24.70*%
R1-D*					***644***
Shannon index	2.97	3.62	3.34	3.46	3.52
ACE index	1853.44	1772.87	2549.92	3510.99	1793.99
Chao1 index	1321.25	1294.05	1635.33	2146.27	1205.94
Coverage	0.99	0.99	0.98	0.98	0.99

	R2				
	R2-A	R2-B	R2-C	R2-D	R2-D*
R2-A	***504***	*17.16*%	*14.75*%	*13.85*%	*15.93*%
R2-B		***534***	*25.47*%	*23.76*%	*29.11*%
R2-C			***476***	*29.04*%	*30.71*%
R2-D				***466***	*36.78*%
R2-D*					***486***
Shannon index	2.45	2.87	2.79	2.72	2.64
ACE index	1452.42	1720.99	1316.17	1200.81	1116.02
Chao1 index	1093.50	1100.14	908.40	898.19	856.92
Coverage	0.99	0.99	0.99	0.99	0.99

	R3				
	R3-A	R3-B	R3-B*	R3-C	R3-C*
R3-A	***473***	*29.84*%	*25.04*%	*32.67*%	*29.45*%
R3-B		***332***	*31.96*%	*36.36*%	*33.14*%
R3-B*			***341***	*28.57*%	*27.83*%
R3-C				***388***	*40.82*%
R3-C*					***371***
Shannon index	2.41	2.20	2.04	2.67	2.50
ACE index	1081.79	770.46	1073.73	872.75	848.48
Chao1 index	824.01	579.69	727.47	750.50	610.42
Coverage	0.99	0.99	0.99	0.99	0.99

Values for unique OTU numbers are bold italic, and those for shared OTUs are italic

### Similarity analysis of all samples

**Fig. 1:**
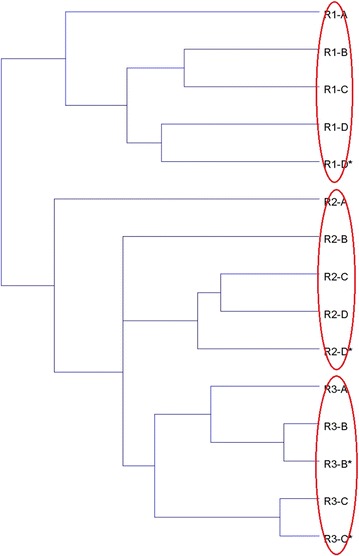
Cluster analysis of all samples at phylum level based on Bray–Curtis distances in three PBS denitrification reactors

**Fig. 2:**
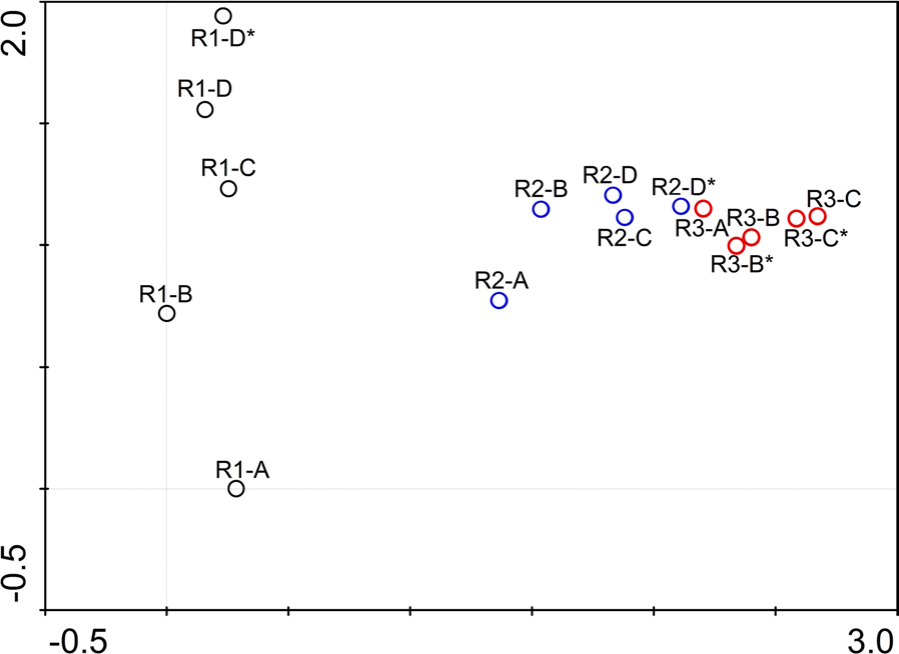
Detrended correspondence analysis (DCA) of all samples based on phylum level

The cluster analysis of all samples was conducted at phylum level and the result was shown in Fig. 1. The results showed that at the phylum level, the samples in Reactor I and Reactor II/III was clustered into two separate groups. In the second group, the samples of Reactor III were also clustered into a same smaller group. Besides, the microbial component of Reactor I and II was of great difference between start-up period using stimulated wastewater (A) and real RAS wastewater (C and D). At the same time point, the two samples taken at different heights of the three reactors were clustered into the same group. Moreover, to identify overall similarities and differences among the three reactors, DCA analysis was also conducted in phylum level (Fig. 2). Similar to the result of CA, in the DCA analysis all the samples were well separated from each other, with partial overlaps in samples from Reactor III which showed more similarity with Reactor II compared to Reactor I.

### The relationships between bacterial communities and operated conditions

**Fig. 3:**
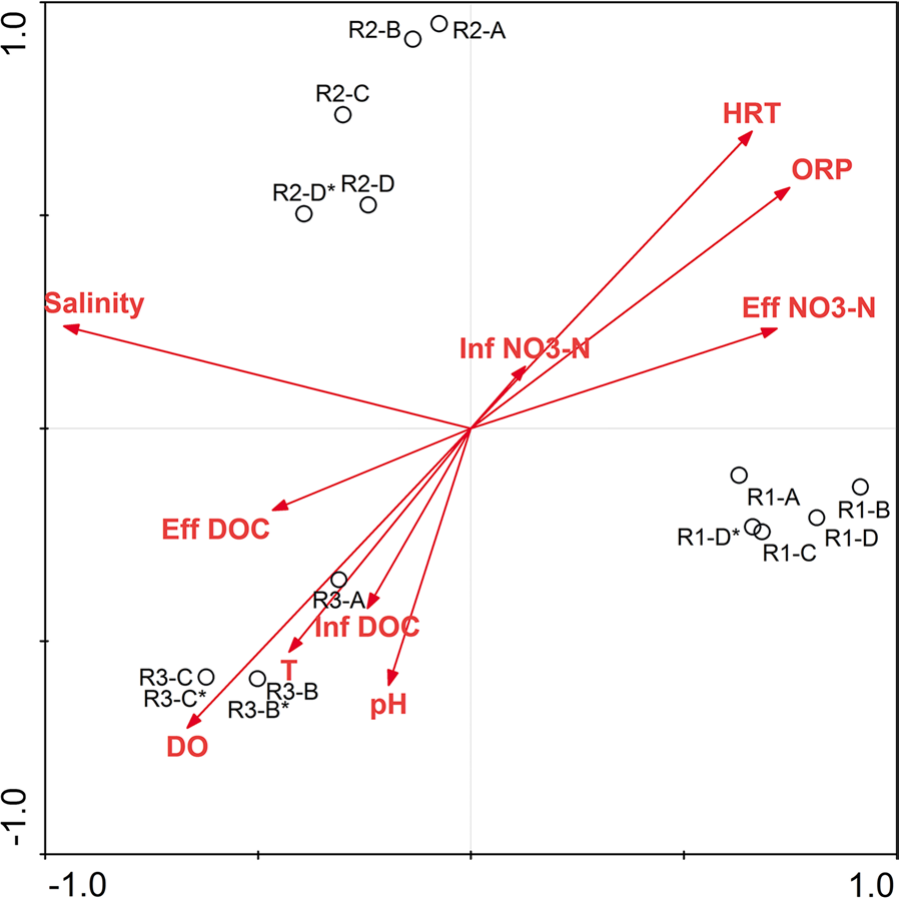
Redundancy analysis (RDA) between sequencing result of genus level and environmental factors

To discern the correlation between the environmental variables and bacterial diversity in the three reactors, ten environmental variables, i.e. salinity, DO, temperature, influent NO_3_-N, effluent NO_3_-N, influent DOC, effluent DOC, influent pH, influent ORP and HRT were selected for RDA analysis. The length of the arrows in the RDA plot corresponds with the strength of the correlation between variables and community structure. It was shown that salinity, ORP, DO and HRT were the four dominating variables influencing the community distribution in denitrification reactors (Fig. 3). Salinity was the most influential factor to distinguish the microbial community between Reactor I and Reactor II/III. Similarly, the difference of bacterial species among Reactor I/II and Reactor III was caused by DO. As the operation of Reactor II, the influence of HRT and ORP became much smaller. Temperature and pH had a positive effect on Reactor III, while negatively affected Reactor I and II. The substrate for denitrification, influent DOC and nitrate, had little influence compared with other environmental factors.

### Abundance of *NosZ* genes and total bacteria among the three PBS denitrification reactors

**Fig. 4:**
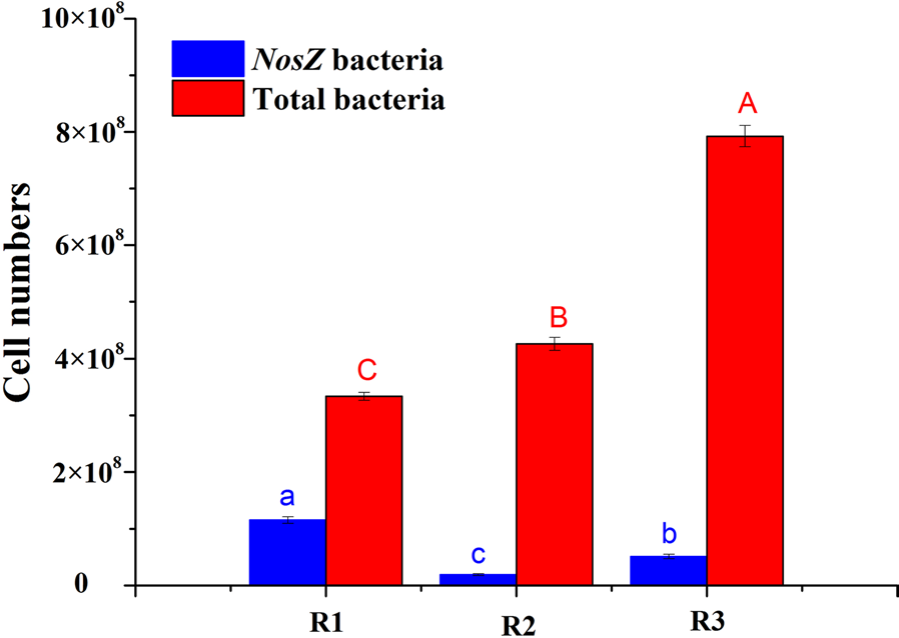
Absolute abundance of *nosZ* and 16 s rRNA cells of all samples using quantitative real-time PCR. Values (mean ± SD) in the *same color* column with *different letters* are significantly different (P < 0.05)

The cell numbers of total bacteria and denitrifiers were shown in Fig. 4 by the quantification analysis of 16 s rRNA and *nosZ* gene, respectively. The *nosZ* gene catalyzing the final step of denitrification was always used to characterize the potential of denitrification capability. Therefore, its proportion in all bacterial community was also calculated. The average bacterial cells of the three reactors were 3.34 × 10^8^, 4.26 × 10^8^ and 7.92 × 10^8^ with the average denitrifiers cells of 1.15 × 10^8^, 0.19 × 10^8^ and 0.51 × 10^8^. Overall, both salinity and DO had significantly increased the bacteria cell number within the three reactors though the denitrifiers were restrained (P < 0.05). As for denitrifiers, the average nosZ gene proportion in Reactor I (salinity, 0‰) was 34.5%, followed by Reactor III and II (salinity, 25‰) which own 6.4 and 4.5%, respectively which demonstrated that DO could stimulate denitrifiers proliferation in salinity reactor. It was obvious to notice that salinity stress has a great negative influence on the distribution of denitrifiers containing *nosZ* gene while DO favored both for bacteria and denitrifiers proliferation.

## Discussion

### Microbial α-diversity analysis

Similar variation trend was observed between OTU numbers and ACE/Chao1 indexes. As for OTU numbers, within all 15 samples, 332–901 OTUs were obtained with an average of 542 OTUs per sample. The diversity and richness of bio-denitrification reactors were lower compared to industry wastewater treatment plant (Ma et al. 2015) and municipal wastewater treatment systems (Xia et al. 2010), which was probably due to the differences in salinity, carbon source and the relatively unitary function that focused on denitrification in our reactors. Besides, Reactor I own the richest OTU numbers followed by Reactor II and Reactor III have the lowest OTU numbers which were consistent with the rarefaction cures of all samples (Additional file 1: Figure S1). This result demonstrated that environmental factors, such as salinity and DO, played as selective pressure, decreased the bacterial diversity in denitrification reactors. This phenomenon that salinity decreasing microbial diversity was also reported by other researchers that focused on granular sludge bed reactor (Miao et al. 2015). However, no obvious negative effect of DO on OTU numbers was detected in this study which was controversial with the report that the biofilm developed in oxygen presence had a smaller bacterial density and a lower denitrifying bacteria versus nitrate reducing bacteria ratio in a submerged filter system using sucrose as carbon source (Gómez et al. 2002). This could explain the hypothesis that biofilm attached to solid-phase carrier can endure certain environmental pressure induced by DO and that DO depletion through respiration can also introduce anoxic micro-zones in the deep layer of biofilm. Moreover, the OTU numbers in marine reactors was relatively stable and showed higher shared OTU percentages than Reactor I, which might explain our previous study that Reactor II owns higher and more stable denitrification activities to resist loading shock (Zhu et al. 2015). On the other hand, the variation of OTU numbers in Reactor I caused by sudden increased temperature on day 138 also resulted in nitrite and nitrate acclimation (Zhu et al. 2015). This result indicated the variation of OTU numbers may cause the shift of bacterial species and further influence the denitrification performance.

During acclimation periods of Reactor I and II, the Shannon index increased gradually, which indicated the colonization and reproduction of the bacteria. Then, when real RAS wastewater was used, the Shannon index decreased in both reactors and the bio-diversities were decreased, which could be explained by the higher abundance of dominating species. It was interesting to notice that all the samples in the reactor bottom have lower Shannon index than that of middle, which indicated the microbial diversity got higher along the reactor column to the middle. We assume that in the anoxic up-flow fixed bed reactors (I and II), the relative higher nitrate and DO concentration in the bottom decreased the bacterial diversity, on the other hand, in interval aeration Reactor (III) the relative low DO concentration in the bottom decreased the bacterial diversity. However, the higher carrying capacity introduced by higher DOC concentration in Reactor II as well as lower microbial diversity put it into a situation where is more susceptible to pathogens invaders or opportunistic bacteria proliferation, which may cause disease to aquatic animals (Attramadal et al. 2012). Overall, salinity, as a selective pressure, significantly decreased the microbial diversity and evenness while DO had little effect and higher bio-diversity was discovered in the middle layer of the reactors.

### Bacterial community composition

The dominate phylum and genus of the three reactors were illustrated in previous studies (Ruan et al. 2016; Zhu et al. 2015). Overall, *Proteobacteria* was the most dominant phylum in all samples among which *Betaproteobacteria* ranked first. Moreover, the portions of *Alphaproteobacteria* and *Gammaproteobacteria* decreased in salinity reactors (II and III). It is known that *Proteobacteria* dominates in the reactors treating high-nitrate wastewater, and many types of denitrifiers are included in the phylum *Proteobacteria* (Liao et al. 2013). It was notice that distinguished microbial diversity and component were detected between Reactor I and Reactor II, while Reactor II had similar genus component with Reactor III. As reported in previous studies, salinity is important in shaping microbial communities at both taxonomic and functional gene levels (Miao et al. 2015; Yoshie et al. 2004).

At genus level, *Acidovorax*, *Simplicispira*, *Comamonas* and *Bradyrhizobium* were the four dominant genera in Reactor I, whereas *Azoarcus*, *Simplicispira*, and *OD1* were the main genera in both Reactor II and Reactor III (Ruan et al. 2016; Zhu et al. 2015). It was interesting to notice that *Simplicispira* was detected in all reactors with high abundance (15.76 ± 13.07%). *Simplicispira* is a specie of denitrifiers that has been widely detected in both sludge system and PCL, PHBV as well as PBS denitrification reactors (Chu and Wang 2013; Khan et al. 2007; Shen et al. 2016). In a continuous aerobic-anaerobic coupled (CAAC) moving bed biofilm reactor (MBBR) *Simplicispira* was detected and it can co-exist with the species capable of heterotrophic nitrification and aerobic denitrification at low temperature (Li et al. 2014; Yao et al. 2013). Moreover, *Simplicispira* members of the *Simplicispira* genus have been isolated from wastewater and identified in nitrate reducing and PHA degrading enrichments (Dawson et al. 2013). Hence, *Simplicispira* is a capable denitrifiers that can be cultivated under various conditions, which worth drawing more attention for the treatment of wastewater contaminated with high nitrate. Denitrifiers including *Comamonas*, *bradyrhizobium*, *Afipia*, *Thermomonas*, *Rhizobium*, *Dechloromonas* and *Oligotropha* were only detected in Reactor I, which means it cannot adapt to salinity and oxygen. *Comamonas* is one of the most abundant microorganisms in biofilm communities driving wastewater treatment and was also found in nitrate removing microbial communities under oxygen-limiting conditions (Wu et al. 2015). *Bradyrhizobium* was reported to perform the complete denitrification pathway process (Bedmar et al. 2005). In this experiment, some microorganisms adapting to high salinity gradually became predominant bacteria including *Azoarcus*, *OD1*, *Oceanicola* and *Labrenzia* which were only detected with the existence of salinity, among which *Azoarcus* and *OD1* can survive under both anoxic and intermittent aeration conditions while *Oceanicola* was detected in aeration environment and *Labrenzia* was widely detected in Reactor II. *Acidovorax* was detected in anoxic reactors (Reactor I and II) which once reported as PHB-degrading, denitrifying strain (Khan and Hiraishi 2001). Besides, *Desulfopila*, sulfate reduction bacteria (SRB), was widely detected in Reactor II (anoxic). But low richness of *Desulfopila* was found in Reactor III (interval aeration), which indicated that DO can efficiently inhibit the SRB activity. However, how bacteria distribute along the depth of biofilm because of substrate penetration (i.e. nitrate or DO) and competition need more advanced technology and further study.

Moreover, the similarity of bacterial community within the three reactors were analyzed by DCA and cluster analysis. The DCA result was consistent with cluster analysis and both of which indicated that salinity played an important role in such PBS denitrification microbial community formation. This result was also corresponding to the former bacterial diversity. Moreover, as the microflora getting mature the distance between neighbor samples became closer (Fig. 2), which means the similarities were getting higher, meanwhile the percentage of shared OTUs getting bigger (Table 1). The relative stable microbial component during the mature period of the experiment may explain the high and steady denitrification activities and performance. Besides, the microbial component of sample R2-D* in the bottom of Reactor II had high similarity with R3-A, which demonstrated that smaller HRT and high influent flow rate brought certain DO existed in real RAS wastewater to the reactor and DO shifted the microbial component in Reactor II to the direction of Reactor III.

Salinity and DO have a great influence on microbial formation and diversity in this study. Studying microbial communities helps us to understand how microbes play essential roles in biogeochemical cycling and ecosystem functioning. Future studies have to include the experiment of replicates to improve the opportunities of explicating the microbial shift dynamics and obtaining planned and desirable microbial communities for environmental engineering purpose.

### Relationship between operation parameters and bacterial community

RDA analysis was performed to demonstrated the relationship between environmental factors and bacterial communities. Among all operation parameters, salinity was the most significant factor for microbial community formation between Reactor I and II/III, which was correspond to DCA and cluster analysis (Figs. 2, 3). Similarly, the microbial community in aerobic granular sequencing batch reactor obviously changed as the increase in salinity (Wang et al. 2015). A study conducted by Miao in expanded granular sludge bed revealed that decrease of influent NaCl concentration from 11 to 0%, the bacterial community have changed greatly at both phylum and genera level and the denitrification performance as well as denitrifying functional genes had increased when the salinity stress disappeared (Miao et al. 2015). Besides, salinity gradient in a coastal aquifer plays an important role in shaping the bacterial assemblages (Santoro et al. 2006). In our study, salinity plays an important role in shaping the microbial community and for further study, the influence of salinity gradients on denitrifiers’ component and activities in one reactor may need more research.

Besides, DO play a determinate role in microbial community under anoxic/aeration condition. Though most of the denitrifying bacteria are facultative anaerobes, simultaneous ammonia and nitrate removal was achieved through precise DO control strategy in Reactor III (Ruan et al. 2016). Denitrification produces less energy yield than oxygen respiration, therefore, a bacterial cell growing in aerobic conditions will choose to use oxygen as terminal electron acceptor and oxygen reversible inhibits the activities of the denitrification enzymes and regulate the denitrification gene expression (Kampschreur et al. 2009). Though most research reported oxygen can decrease denitrification rate, many investigators had found that very low DO concentrations could cause complete cessation of denitrifying activity by certain strains belonged to *Comamonadaceae*, *Brevundimonas* and *Acidovorax* (Chen et al. 2012; Huang et al. 2013).

As most research proved that influent nitrate loading also had great influence on denitrification performance and microbial diversity. It was worthy to note that HRT had more influence than influent nitrate concentration. Our RDA analysis proved previous study of PCL denitrification reactor that HRT obtained more potential to affect the nitrate removal rate than influence nitrate concentration under the same influent nitrate loading (Shen et al. 2013). First, different HRT produces varied shear force, thus influence the biofilm thickness and mass transfer. What’s more, HRT is an important factor controlling the release and utility of DOC, which also changed the microbial structure. However, as the biofilm getting thick and mature, the influence of influent nitrate loading became less important in Reactor I and II. It demonstrated the fact that compared to liquid denitrification system, biofilms attached to biodegradable polymers can resist certain influent pressure, which make it more applicable for nitrate removal in RAS production system. Compared to other factors, influent DOC seemed to have less effect on biofilm composition. This might because the DOC can be released from PBS automatically by enzyme degradation according to the nitrate loading and DOC was not a limiting factor in this experiment. Since most proportion of dissolved carbon source used for denitrification was from PBS biodegradation, the passive diffusion mode requires less DOC monitoring and saves the management cost.

### The abundance of bacteria containing 16S rRNA and *nosZ* genes

Since such environmental variables as temperature and influent type were identical for all three reactors in the last sample time (Additional file 1: Table S1, R1-D*, R2-D*, R3-C*), the effect of salinity and DO on denitrifiers distribution in total bacteria cells could be demonstrated using quantitative PCR, which was shown in Fig. 4. As explained in previous study, salinity was the key determinant of *nosZ* community composition in the coastal wetland soil environment (Zhe et al. 2012), the bacterial cells containing *nosZ* gene were significantly lower in the reactors with salinity compared with Reactor I. The lower *nosZ* copies were consistent with previous study reported that salinity decreased the biodiversity of bacteria carrying the denitrification genes (Miao et al. 2015). Besides, investigation of the diversity of *nirK* and *nirS* in denitrifying bacteria revealed that salinity decreased the diversity in a nitrate-containing saline wastewater treatment system (Yoshie et al. 2004). How it is interesting to notice that salinity have increased the overall bacterial population, which could be explain by the higher DOC concentration in Reactor II (Zhu et al. 2015). In RAS system, it is believed that the carrying capacity is defined by density dependent restrictions like availability of resources, which typically is supply of dissolved organic matter (DOM) for the heterotrophic bacteria (Attramadal et al. 2012). Hence higher DOC release in salt water resulted in higher bacteria population.

Unexpectedly, Reactor III displayed a higher denitrifier and bacterial cell number compared to Reactor II, but we can hardly draw the conclusion that DO can both significantly improve the total bacteria and denitrifiers population in denitrification reactor since the influent nitrate loading of Reactor III was much higher than that of Reactor II (Additional file 1: Table S1), which could also be a reason for higher bacteria population. To be noticed, the *nosZ* portion of all bacteria under interval aeration condition was similar with anoxic condition, which at least proved the feasibility to perform denitrification with oxygen existence which can save the cost for oxygen degassing of oxygen in denitrification unit. Another explanation could be that aeration improved the substance transcription and harbor higher denitrification activities. Similarly, the expression of *nosZ* under suboxic and oxic conditions was also evidenced in marine environments, and was attributed to the generation of O_2_-depleted microzones inside bacterial aggregates (Wyman et al. 2013).

Overall, Reactor I had a higher denitrifiers share, it could be inferred that it might have more potential in denitrification, but the short of exploitable DOC might be a restrict factor. Besides, no obvious influence of DO on *nosZ* gene expression was observed which worth more attention and rigorous experiment design to explicit its effect on DOC release and denitrification genes expression. Furthermore, comprehensive study of the relationships among qualitative and quantitative microbial community compositions, functions, and process stabilities are needed which will help in the design of advanced wastewater treatment systems or determination of appropriate operational conditions.

## Additional file

**Additional file 1: Table S1.** The operation conditions of three reactors and DNA sample time. **Table S2.** Primers and conditions for quantitative real-time PCR in this study. **Figure S1.** Richness rarefaction curves of all samples.

## Abbreviations

RAS: recirculating aquaculture system
BDPs: biodegradable polymers
PHAs: polyhydroxyalkanoates
PCL: polycaprolactone
PLA: polylactic acid
PBS: poly(butylene succinate)
DO: dissolved oxygen
HRT: hydraulic retention time
PHB: polyhydroxybutyrate
DOC: dissolved organic carbon
OTUs: operational taxonomic units
RDP: ribosomal database project
ACE: abundance-based coverage estimator
CA: cluster analysis
PCA: principal component analysis
DCA: detrended correspondence analysis
RDA: redundancy analysis
CAAC: continuous aerobic-anaerobic coupled
MBBR: moving bed biofilm reactor
SRB: sulfate reduction bacteria
DOM: dissolved organic matter
