# Supplementary material for: The impact of DO and salinity on microbial community in poly(butylene succinate) denitrification reactors for recirculating aquaculture system wastewater treatment
Source: AMB Express. 2017 Jun 2;7:113. doi: 10.1186/s13568-017-0412-3 (PMC5457379; doi:10.1186/s13568-017-0412-3)

AMB express

Supplementary materials

The impact of DO and salinity on microbial community in poly(butylene succinate) denitrification reactors for recirculating aquaculture system wastewater treatment

Ya-Le Deng[1,2], Yun-Jie Ruan[1, 3], Song-Ming Zhu[1], Xi-Shan Guo[1], Zhi-Ying Han[1], Zhang-Ying Ye[1], Gang Liu[1], Ming-Ming Shi[1]

1. Institute of Agricultural Bio-Environmental Engineering, College of Bio-systems Engineering and Food Science, Zhejiang University, Hangzhou, 310058, China; 2. Aquaculture and Fisheries Group, Department of Animal Sciences, Wageningen University, 6708 WD Wageningen, The Netherlands; 3. Department of Biological and Environmental Engineering, Cornell University, Riley Robb Hall, Ithaca, NY 14853, USA

*Corresponding author: Yun-Jie Ruan, Zhejiang University, 866 Yuhangtang Road, Hangzhou, China 310058,

Phone: +86-571-88982373

Email: ruanyj@zju.edu.cn; ry85@cornell.edu

Additional file 1:

Table S1. The operation conditions of three reactors and DNA sample time

Operation mode	HRT (h)	Influent type	Influent nitrate loading (kg NO_3^--N m^{-3} d^{-1})	Temperature (ºC)	Time (d)	Sample number[a]
anoxic	8	Synthetic RAS wastewater	0.08±0.01	19±1	13	R1-A
						R2-A
					50	R1-B
						R2-B
		Real RAS wastewater	0.19±0.09		110	R1-C
						R2-C
			0.22±0.02	24±1	138	R1-D
						R1-D*
						R2-D
						R2-D*
anoxic/oxic	5		0.64±0.20		45	R3-A
					75	R3-B
						R3-B*
					100	R3-C
						R3-C*

Note:

[a] R1, Reactor I (salinity, 0‰); R2, Reactor II (salinity, 25‰); R3, Reactor III (salinity, 25‰); * was collected in the bottom while others was collected in the middle of the reactors.

Additional file 1:
Table S2. Primers and conditions for quantitative real-time PCR in this study

Bacterial Group	Targeted Gen	Primers Name	Sequence (5'-3')	References
Bacteria	V3-16S rRNA	1369F	CGGTGAATACGTTCYCGG	(Suzuki et al. 2000)
		1492R	GGWTACCTTGTTACGACTT	
Denitrifiers	*nosZ*	Nos1527F	CGCTGTTCHTCGACAGYCA	(Scala and Kerkhof 1998)
		Nos1773R	ATRTCGATCARCTGBTCGTT	

Target Gene	Initial Denaturalization	Denaturalization	Primers Annealing	Elongation	Final Extension
nosZ	95ºC 10 min	95ºC 10 sec	60ºC 1 min	60ºC 1 min	72 ºC 7min
V3-16S rRNA	95ºC 10 min	95ºC 1 min	55ºC 1 min	72ºC 2 min	72 ºC 7min

References

Scala DJ, Kerkhof LJ (1998) Nitrous oxide reductase (nosZ) gene-specific PCR primers for detection of denitrifiers and three nosZ genes from marine sediments Fems Microbiol Lett 162:61-68 doi:DOI 10.1111/j.1574-6968.1998.tb12979.x

Suzuki MT, Taylor LT, DeLong EF (2000) Quantitative analysis of small-subunit rRNA genes in mixed microbial populations via 5 '-nuclease assays Appl Environ Microb 66:4605-4614

Additional file 1:
Figure S1. Richness rarefaction curves of all samples.

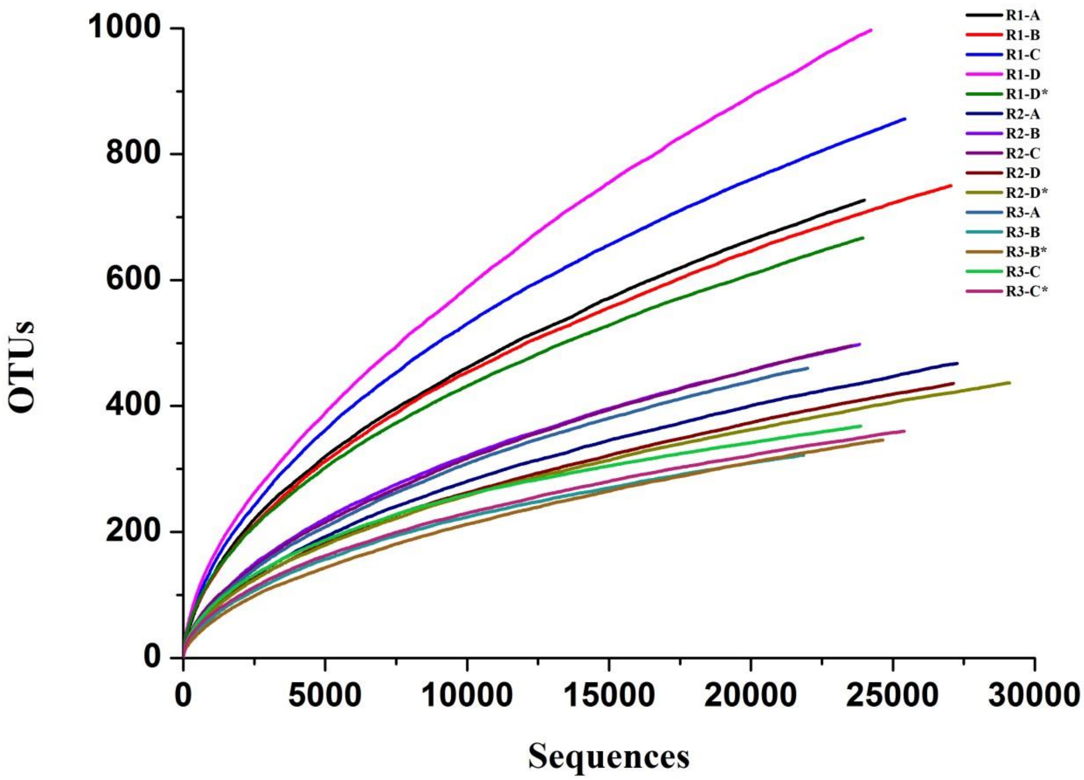

Supplement: Supplementary file 1 — Additional file 1: Table S1. The operation conditions of three reactors and DNA sample time. Table S2. Primers and conditions for quantitative real-time PCR in this study. Figure S1. Richness rarefaction curves of all samples. [file 13568_2017_412_MOESM1_ESM.pdf]
